# Comparative study of the sensitivity of ultra-high-resolution CT and high-resolution CT in the diagnosis of isolated fenestral otosclerosis

**DOI:** 10.1186/s13244-023-01562-y

**Published:** 2023-11-28

**Authors:** Ning Xu, Heyu Ding, Ruowei Tang, Xiaoshuai Li, Zhengyu Zhang, Han Lv, Chihang Dai, Xiaoyu Qiu, Yan Huang, Xu Han, Guo-Peng Wang, Yuhe Liu, Shusheng Gong, Zhenghan Yang, Zhenchang Wang, Pengfei Zhao

**Affiliations:** 1 Department of Radiology, Beijing Friendship Hospital, Capital Medical University, No. 95, Yongan Road, Xicheng District, Beijing, 100050 China; 2 Department of Otorhinolaryngology Head and Neck Surgery, Beijing Friendship Hospital, Capital Medical University, No. 95, Yongan Road, Xicheng District, Beijing, 100050 China

**Keywords:** Otosclerosis, Fissula ante fenestram, Sensitivity, Tomography (x-ray computed), Resolution

## Abstract

**Purpose:**

To compare the diagnostic sensitivity of ultra-high-resolution computed tomography (U-HRCT) and HRCT in isolated fenestral otosclerosis (IFO).

**Methods:**

A retrospective analysis was conducted on 85 patients (85 ears) diagnosed with IFO between October 2020 and November 2022. U-HRCT (0.1 mm thickness) was performed for 20 ears, HRCT (0.67 mm thickness) for 45 ears, and both for 20 ears. The images were evaluated by general radiologists and neuroradiologists who were blinded to the diagnosis and surgical information. The diagnostic sensitivity of U-HRCT and HRCT for detecting IFO was compared between the two groups.

**Results:**

Excellent inter-observer agreement existed between the two neuroradiologists (Cohen’s *κ* coefficient 0.806, 95% CI 0.692–0.920), with good agreement between the general radiologists (Cohen’s *κ* coefficient 0.680, 95% CI 0.417–0.943). U-HRCT had a sensitivity of 100% (40/40 ears) for neuroradiologists and 87.5% (35/40 ears) for general radiologists, significantly higher than HRCT (89.2% [58/65 ears] for neuroradiologists; 41.5% [27/65 ears] for general radiologists) (*p* = 0.042, *p′* < 0.000). General radiologists’ sensitivity with HRCT was significantly lower compared to neuroradiologists (*p* < 0.000), but no significant difference was observed when general radiologists switched to U-HRCT (*p* = 0.152). Among the 20 ears that underwent both examinations, U-HRCT detected lesions smaller than 1 mm in 5 ears, whereas HRCT’s sensitivity for neuroradiologists was 40% (2/5 ears), significantly lower than for lesions larger than 1 mm (93.3%, 14/15 ears, *p* = 0.032).

**Conclusion:**

U-HRCT exhibits higher sensitivity than HRCT in diagnosing IFO, suggesting its potential as a screening tool for suspected otosclerosis patients.

**Critical relevance statement:**

Ultra-high-resolution computed tomography has the potential to become a screening tool in patients with suspected otosclerosis and to bridge the diagnostic accuracy gap between general radiologists and neuroradiologists.

**Key points:**

• U-HRCT exhibits higher sensitivity than HRCT in the diagnosis of IFO.

• U-HRCT has a significant advantage in the detection of less than 1 mm IFO.

• U-HRCT has the potential to be used for screening of patients with suspected otosclerosis.

**Graphical Abstract:**

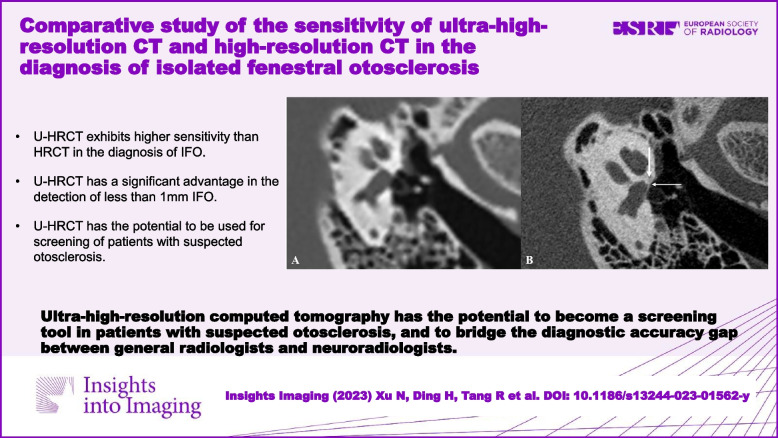

## Introduction

Otosclerosis, a primary focal osteodystrophy of the otic capsule [1], is one of the leading causes of acquired hearing loss in adults, with an estimated prevalence of approximately 0.3 to 1% [2]. This condition is typically characterized by conductive hearing loss [3], and up to one-third of patients may eventually develop mixed hearing loss [4]. Sensorineural hearing loss, while possible, is comparatively rare. Otosclerosis is commonly categorized into two types based on the location of occurrence: fenestral and retrofenestral, with the former being substantially more common [5]. In terms of pathological manifestations, otosclerosis can be subdivided into the otospongiotic and the otosclerotic phases.

The diagnosis of otosclerosis predominantly hinges on a combination of clinical symptoms, audiological assessments, and intraoperative observations. Classic clinical symptoms include bilateral progressive hearing loss in the absence of tympanic membrane abnormality, with some patients also presenting a family history; an audiological examination often reveals an air–bone gap with Cahart’s notch and the absence of the stapedius reflex; intraoperative observations demonstrate stapes fixation without any ossicle deformities. However, it is noteworthy that the full spectrum of these clinical and audiological characteristics is not often present, only occurring in approximately 7.5 to 35% of cases [6, 7]. Thus, clinicians must exercise discretion in diagnosing otosclerosis, even in the absence of the full array of symptoms.

High-resolution computed tomography (HRCT) holds significant value in differential diagnosis, surgical planning, prognostic assessment, and surgical failure analysis for otosclerosis patients [8]; nevertheless, there’s no firm consensus regarding the routine application of HRCT for assessing patients with clinically suspected otosclerosis [7, 8]. Indeed, it is widely acknowledged that HRCT exhibits high specificity and positive predictive value when diagnosing otosclerosis [6, 9–11]. The diagnostic sensitivity of HRCT for otosclerosis, as reported, exhibits wide variability, ranging from 10 to 100% [6, 12, 13]. In the context of some reports, when compared to clinical history and audiological examination, the diagnostic sensitivity of HRCT does not markedly improve the detection of otosclerosis, particularly in cases of isolated fenestral otosclerosis (IFO) without retrofenestral lesions [7, 14].

The variable diagnostic sensitivity of HRCT could likely be attributable to general radiologists potentially overlooking subtle lesions or varying degrees of sclerosis, due to the poor contrast with the surrounding structures. Besides, the thickness of the conventional HRCT is mostly 0.6 mm. This spatial resolution, however, is insufficient to delineate typical IFO smaller than 1 mm [8, 12].

Improvements in CT resolution, such as in newly reported photon-counting computed tomography (PCCT) with a spatial resolution of up to 0.2 mm, have enabled clear observation of minute temporal bone structures and fenestral otosclerosis, as demonstrated by Benson et al. [15]. Our team has developed ultra-high-resolution computed tomography (U-HRCT) with an even higher resolution of up to 0.05 mm, based on cone beam computed tomography (CBCT) principles. U-HRCT excels in delineating fine anatomical structures of the temporal bone [16] and has the potential to further enhance the detection of concealed lesions [5, 17]. However, clinical studies comparing U-HRCT’s diagnostic efficiency for temporal bone lesions with HRCT are currently lacking. and its comparative advantages in a clinical setting remain unknown.

Considering this, we retrospectively analyzed the data of patients with surgically confirmed IFO and compared the diagnostic sensitivity of general radiologists and neuroradiologists in diagnosing IFO using U-HRCT and HRCT. The main objective was to assess the utility of U-HRCT in screening for IFO.

## Materials and methods

### Patients

This retrospective study was performed at our tertiary center, approved by the local ethical committee (IRB: 2022-P2-055–01, 2020-P2-061–01), with written informed consent obtained from all patients. The patients included in this study sought medical attention at the Department of Otorhinolaryngology Head and Neck. The decision of whether to perform HRCT or U-HRCT was made through a collaborative determination between the clinicians and the patients after thorough communication. Throughout the scanning process, we strictly adhered to ethical requirements, ensuring patient privacy protection and minimizing radiation exposure.

The inclusion criteria were as follows: (1) patients hospitalized due to suspected otosclerosis between October 2020 and November 2022; (2) no history of chronic otitis media or mastoiditis on the affected side, ear surgery, trauma, or other related ear conditions; (3) pure tone audiometry finding of an air–bone gap more than 10 dB; (4) preoperative HRCT and/or U-HRCT examination at our hospital; (5) stapedotomy was performed with the detailed plan as previously described [18], and the fixation of the stapes and the movement of the malleus and incus were observed intraoperatively; and (6) postoperative hearing significantly improved. The exclusion criteria included the following: (1) CT evidence of middle ear mastoiditis, cholesteatoma, or tympanic sclerosis; (2) malformation of ossicles or suspected congenital stapes fixation on CT images; (3) retrofenestral otosclerosis on CT images; and (4) for patients who underwent both U-HRCT and HRCT, there was an interval of more than 6 months between the two examinations.

**Fig. 1:**
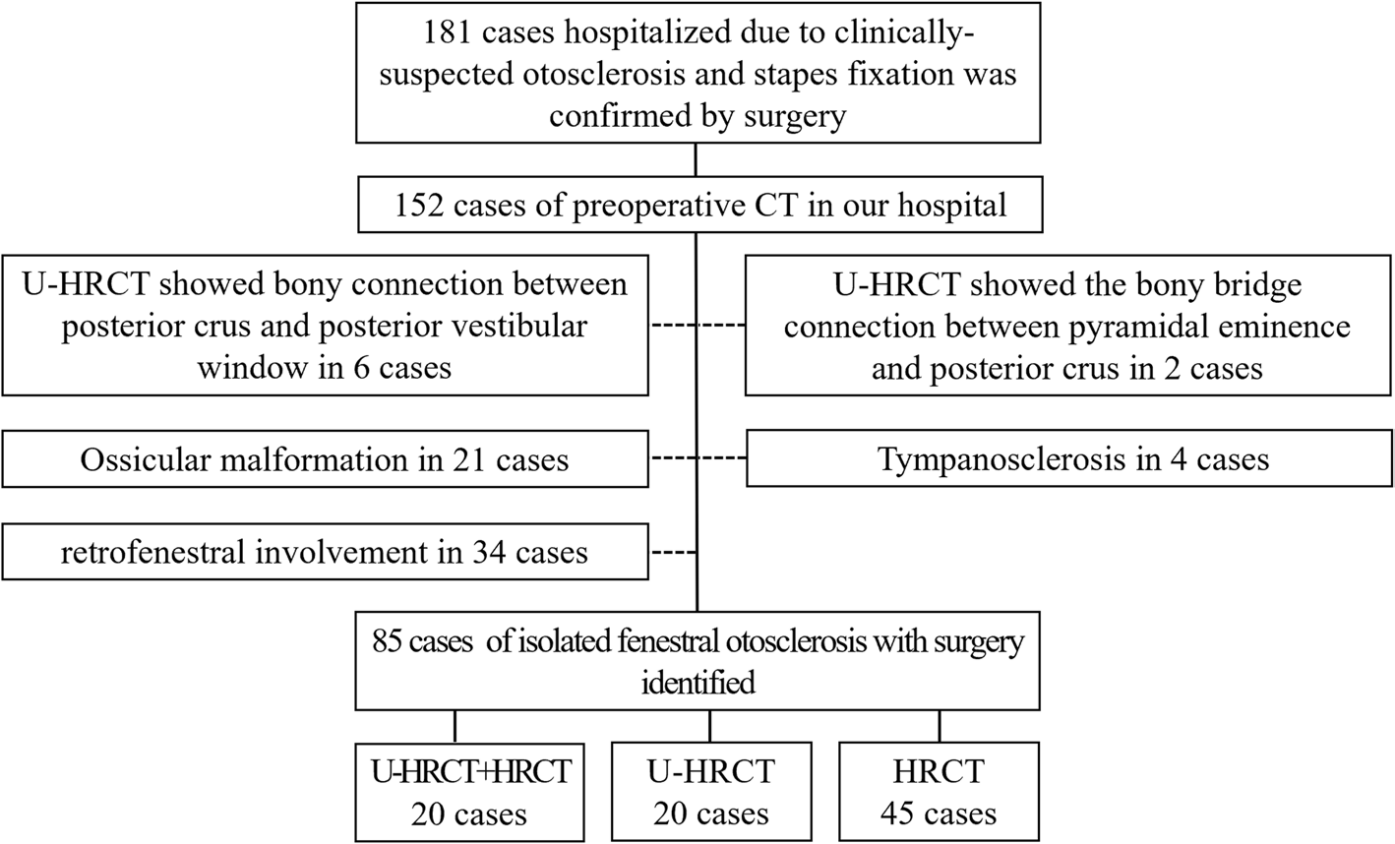
Inclusion process and diagnostic results of research objects

There were 255 ears that showed signs of otosclerosis on CT. Among them, 181 underwent surgery, and 74 did not. Finally, a total of 85 patients (85 ears) with IFO were included, including 48 right operated ears and 37 left operated ears. There were 60 females and 25 males, with an average age of 42.3 ± 12.0 years. Twenty ears underwent both U-HRCT and HRCT, while 20 ears underwent only U-HRCT, and 45 ears underwent only HRCT (Fig. 1).

### Methods

#### Image acquisition U-HRCT

Patients were scanned using an ultra-high-resolution CT scanner (Ultra3D, LargeV). The scanning range was from the apex of the petrous bone to the mastoid tip. The parameters were set as follows: 100–110 kVp; 120–180 mAs; field of view, 65 mm × 65 mm; and isotropic 0.1 mm. The scan comprised 370 layers, and the exposure time was 20 s for each side.

##### HRCT

Patients were scanned using either a 64-channel CT scanner (Brilliance, Philips Healthcare) or a 256-channel CT scanner (Revolution, GE Healthcare). The acquisition parameters were as follows: 100–140 kV; 120–200 mA; matrix, 512 × 512; field of view, 180–220 mm × 180–220 mm; collimation, 16 or 64 × 0.625; slice thickness, 0.67 mm; slice spacing, 0.33 mm; pitch, 0.6 mm; and bone algorithm reconstruction.

#### Diagnostic criteria

**Fig. 2:**
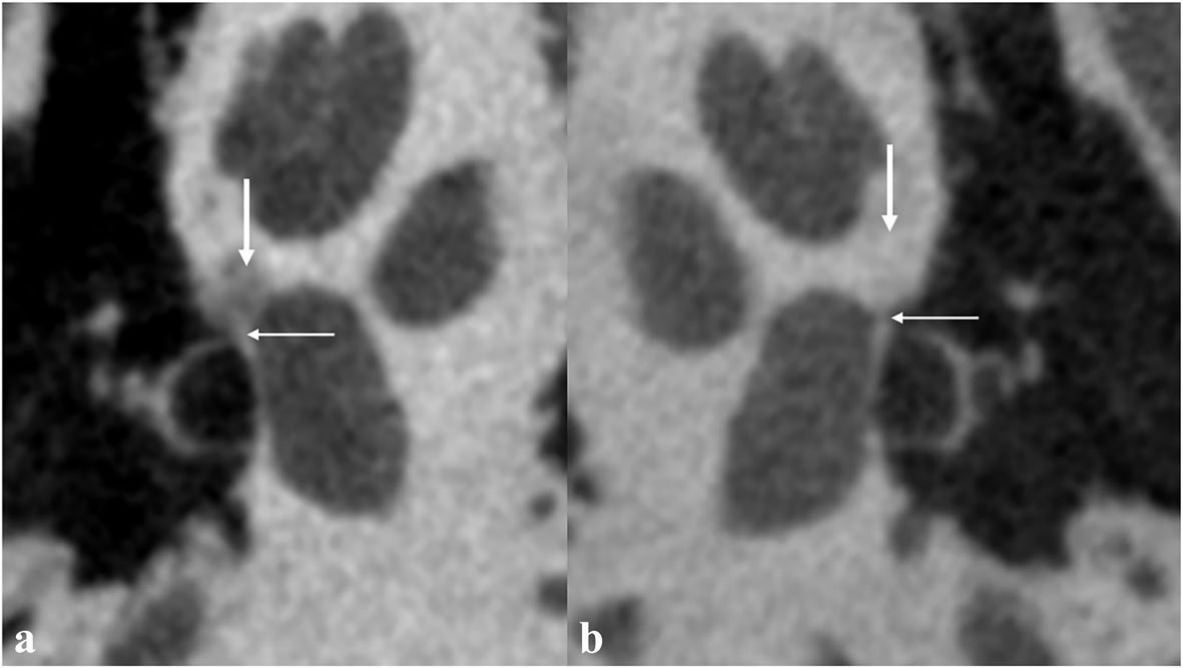
Typical U-HRCT imaging findings of fenestral otosclerosis in the otospongiotic phase. **a** Decreased bone density in the right fissula ante fenestram (thick arrow) and thickening of the right annular ligament (thin arrow). **b** Uniform bone density in the left fissula ante fenestram (thick arrow), and the left annular ligament is clearly shown with linear soft tissue density (thin arrow)

**Fig. 3:**
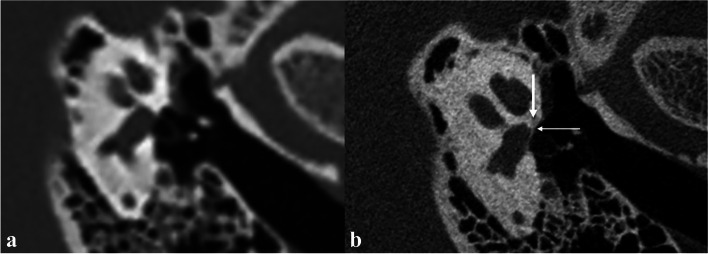
Typical imaging findings of fenestral otosclerosis in the otosclerotic phase. HRCT (**a**) shows no definite positive signs. U-HRCT (**b**) shows no obvious reduction in the density of fissula ante fenestram (thick arrow), increased density of adjacent annular ligament, and slightly thick stapes footplate (fine arrow)

Fenestral otosclerosis was defined by the occurrence of the foci at the external wall of the otic capsule with the fissula ante fenestram, the round window, the oval window, or the facial canal involved. Retrofenestral otosclerosis was defined when the foci were located more medially within the otic capsule [8, 19]. IFO was defined as the presence of fenestral otosclerosis without concurrent retrofenestral involvement. The otospongiotic phase was diagnosed by a notable reduction in bone density (Fig. 2). The otosclerotic phase was diagnosed when the density of the foci increased, making it challenging to differentiate from the normal otic caupusle; this phase was further defined by irregular shapes in the corresponding sites (Fig. 3) and an otic capsule thickness exceeding 2.3 mm [8, 19].

#### Imaging analysis

**Fig. 4:**
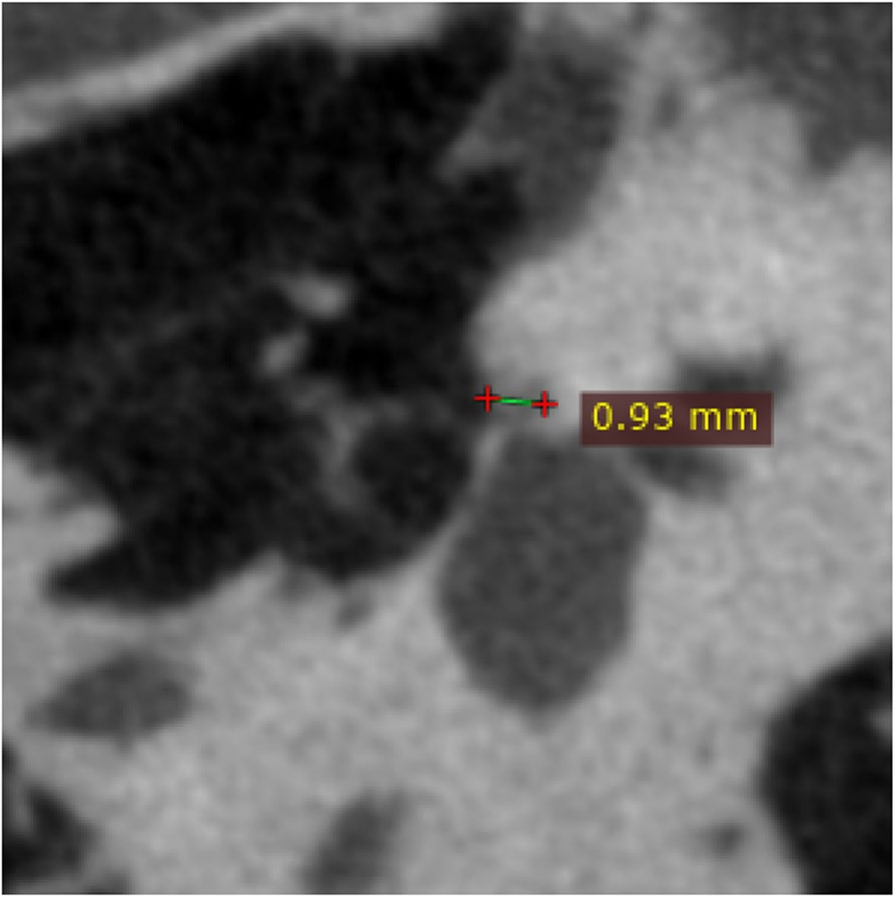
Fenestral otosclerosis. The maximum diameter of the lesion is 0.93 mm

The original HRCT and U-HRCT images were imported into RadiAnt DICOM Viewer for multiplaner reconstruction. This included standardized axial/coronal images, reconstructed parallel/perpendicular to the horizontal semicircular canals, as well as double oblique reformations of the stapes. The reconstructed images maintained the original slice thickness. The maximum diameter of the lesion was measured at the double oblique reformations of the stapes (Fig. 4).

Two general radiologists, with 8 and 12 years of experience respectively, independently evaluated the images. Multiplanar reconstructions were performed using the original images by the radiologists themselves. In the event of a discrepancy, consensus was reached through discussion. Simultaneously, two neuroradiologists, with 8 and 13 years of experience respectively, also evaluated the images, resolving any disagreements through consensus. All radiologists were aware of the main clinical symptom-hearing loss but were kept blind to the side of the symptom, the side chosen for surgery, and intraoperative findings. All radiologists reviewed images from both sides, but only the data corresponding to the surgical side were used for statistical analysis.

#### Statistical methods

Statistical analysis was performed using the SPSS 19.0 software. Cohen’s kappa test was employed to measure the level of agreement between each two independent observers, with the strength of agreement interpreted as follows: slight (0.00–0.20), fair (0.21–0.40), moderate (0.41–0.60), good (0.61–0.80), and excellent (0.81–1.00). Differences between the groups were assessed with the chi-squared test. A *p* value of less than 0.05 was considered statistically significant.

## Results

There were no significant differences between the HRCT group and the U-HRCT group in terms of sex, age, laterality, or disease duration. The inter-observer agreement between the two neuroradiologists was excellent, with Cohen’s *κ* coefficient of 0.806 (95% CI 0.692–0.920). Meanwhile, the agreement between the two general radiologists was good, as reflected by Cohen’s *κ* coefficient of 0.680 (95% CI 0.417–0.943).

When evaluated by neuroradiologists, U-HRCT showed a 100% (40/40) sensitivity for the diagnosis of IFO compared to 89.2% (58/65) of HRCT (*p* = 0.042). When evaluated by general radiologists, the sensitivity of U-HRCT for the diagnosis of IFO was significantly higher at 87.5% (35/40) compared to HRCT, which was at 41.5% (27/65) (*p* < 0.000).

Based on HRCT, the sensitivity of general radiologists for diagnosing IFO was significantly lower than that of neuroradiologists (*p* < 0.000). Interestingly, when using U-HRCT, the sensitivity of general radiologists was as good as that of neuroradiologists using HRCT (*p* = 0.152) (Table 1).

**Table 1:** Diagnostic sensitivity of HRCT and U-HRCT when evaluated by general radiologists and neuroradiologists

	Diagnositic sensitivity of HRCT	Diagnositic sensitivity of U-HRCT	*p* value
General radiologists	41.5%	87.5%	0.000
Neuroradiologists	89.2%	100%	0.042
*p* value	0.000	0.152	

Among the 20 patients who underwent both HRCT and U-HRCT, lesions smaller than 1 mm were detected in 5 cases via U-HRCT by neuroradiologists. Out of these five cases, two cases were displayed on HRCT, and three were not. The sensitivity of HRCT was 40% (2/5) in diagnosing lesions less than 1 mm. Meanwhile, the other 15 cases presented with lesions larger than 1 mm on U-HRCT, of which 93.3% (14/15) were found via HRCT by neuroradiologists. The sensitivity of HRCT for diagnosing IFO with lesions smaller than 1 mm was significantly lower than for lesions larger than 1 mm by neuroradiologists (*p* = 0.032).

## Discussion

Our study revealed that U-HRCT achieved a remarkable diagnostic sensitivity of 100% in identifying IFO, surpassing HRCT’s sensitivity for both neuroradiologists and general radiologists. Furthermore, the diagnostic sensitivity of IFO was higher among neuroradiologists compared to general radiologists. However, when general radiologists utilized U-HRCT, their diagnostic sensitivity improved and approached the level of neuroradiologists using HRCT. Notably, we observed that HRCT had a significantly lower sensitivity in detecting IFO smaller than 1 mm compared to lesions exceeding 1 mm. In contrast, U-HRCT demonstrated the ability to clearly visualize and detect lesions smaller than 1 mm.

HRCT has been widely used in the diagnosis of otosclerosis and has a broad range of established indications, including differential diagnosis, staging, prognosis, surgical planning, prediction of postoperative results, and management of complications [8]. However, the diagnostic value of routine imaging studies for otosclerosis remains a topic of debate. The reported sensitivity of HRCT varies across studies due to the differences in patient selection criteria and the expertise of radiologists involved. For instance, Kanona et al. [12] reported that general radiologists identified otosclerosis in only 10% of cases, while neuroradiologists achieved a sensitivity of 100%. Similarly, Maxwell et al. [7] found that general radiologists detected otosclerosis in 29.4% of cases, whereas neuroradiologists achieved a sensitivity of 47.1% [7].

CBCT is widely used in dentistry, but its application in otosclerosis has been limited [8]. Liktor et al. found a sensitivity of 65.62% when comparing CBCT with histopathological results. Notably, CBCT showed a sensitivity of 100% in cases of active otosclerosis but dropped to 0% in cases of inactive otosclerosis [20]. Another study by Redfors et al. found that HRCT had higher sensitivity than CBCT, specifically for fenestral otosclerotic lesions [21]. The U-HRCT device in this study is designed based on the principle of CBCT, and the acquisition layer thickness was 0.1 mm. We had previously utilized this device for stapes imaging and demonstrated superior results compared to HRCT in all aspects [17]. In this study, we present the first application of this device in patients with IFO. The results reveal a remarkable sensitivity of up to 100%. The device enables the delineation of small lesions measuring less than 1 mm and aids in the detection of annular ligament invasion. Furthermore, it provides clear visualization of subtle pathological changes associated with otosclerosis, promoting consistency in diagnoses across radiologists with varying levels of expertise. Therefore, the instrument holds promising potential as a screening tool for otosclerosis and other osseous ear diseases.

Photon-counting computed tomography is an emerging technology in CT that uses photon-counting detectors to count the number of incoming photons and measure photon energy [22]. Early studies have shown that PCCT has lower image noise, thinner slices (0.2 mm), and up to 85% reduction in radiation dose compared to multi-detector computed tomography (MDCT) temporal bone scans [23–25]. Zhou et al. [25] showed that PCCT can achieve an approximately 50% dose reduction compared to MDCT in images of ten cadaveric temporal bone specimens, while maintaining comparable image quality and diagnostic performance. Benson et al. [15] applied PCCT to display the temporal bone anatomy and lesions, and the study showed that PCCT can clearly show the anatomical relationship between the anterior crus of the stapes and the otosclerosis lesion. Rajendran et al. [26] combined a patient’s phantom and technical measurements with clinical measurements for temporal bone imaging. Patient dose measurements found that PCCT reduced the radiation dose by 37% and image noise by 46%. Hermans et al. [27] independently scored the visibility of 14 normal anatomical structures in 36 MDCT and 35 PCCT images of temporal bones without pathology. The results indicated that PCCT provides a better temporal bone anatomical description than MDCT at a lower radiation dose. There is no study on the sensitivity of PCCT for the diagnosis of otosclerosis. PCCT and the ultra-high-resolution CT used in this study have different types of detectors and principles, but both can achieve ultra-high resolution. The CBCT used in this study has twice the signal-to-noise ratio as the conventional MDCT, and the radiation dose is only one-third of the conventional MDCT [28].

Otosclerosis can be classified into two subtypes: fenestral type and retrofenestral type, based on its location. The fenestral type lesions are limited and often not easily detectable on imaging, making them prone to being missed [29]. On the other hand, the retrofenestral type is characterized by abnormal bone density surrounding the cochlea, typically presenting as a bicyclic sign, which is relatively easier to diagnose on imaging. Previous studies investigating the sensitivity of HRCT in diagnosing otosclerosis usually did not differentiate between these two subtypes. Therefore, the present study specifically evaluated the sensitivity of HRCT in diagnosing IFO. The results revealed a significant difference in the sensitivity of HRCT when evaluated by neuroradiologists compared to general radiologists, which is consistent with similar studies conducted internationally [7, 12].

The sensitivity of HRCT assessed by neuroradiologists was found to be 89.2%, indicating that HRCT remains valuable in the diagnosis of otosclerosis. However, the sensitivity of HRCT when examined by routine reporting radiologists was only 41.5%. This finding partially explains why HRCT is in debate as a screening tool for otosclerosis. It is speculated that the variations in subtypes and disease severity among the included patients, along with the differing levels of expertise of the two groups of radiologists, may contribute to the substantial differences in HRCT sensitivity for diagnosing otosclerosis.

Some limitations of this study should be acknowledged. Firstly, 20 patients underwent both HRCT and U-HRCT, and most of these patients were initially misdiagnosed on HRCT. This specific selection of cases may introduce a potential bias. Secondly, the absence of normal controls and the evaluators’ knowledge of patients’ complaint as hearing loss could potentially influence the results. Thirdly, this study was conducted at a single center, which further restricts the generalizability of the findings. Future studies should involve larger multi-center cohorts to obtain more robust and representative evidence.

## Conclusion

U-HRCT demonstrated significantly higher sensitivity than HRCT in diagnosing IFO for both general radiologists and neuroradiologists, highlighting its potential for being used as a screening tool in patients with suspected otosclerosis. Additionally, U-HRCT has the potential to bridge the diagnostic accuracy gap between general radiologists and neuroradiologists.

## Data Availability

Not applicable.

## Abbreviations

HRCT: High-resolution computed tomography
IFO: Isolated fenestral otosclerosis
U-HRCT: Ultra-high-resolution computed tomography
